# Identification of a CpG-based signature coupled with gene expression as prognostic indicators for melanoma: a preliminary study

**DOI:** 10.1038/s41598-023-50614-2

**Published:** 2024-03-04

**Authors:** Zhen Lin, Liu Yang

**Affiliations:** Cancer Center, Department of Medical Oncology, Zhejiang Provincial People’s Hospital (Affiliated People’s Hospital), Hangzhou Medical College, Hangzhou, Zhejiang China

**Keywords:** Tumour biomarkers, Cancer epigenetics

## Abstract

DNA methylation is an important part of the genomic biology, which recently allowed the identification of key biomarkers for a variety of cancers, including cutaneous melanoma. Despite the current knowledge in cutaneous melanoma, there is a clear need for new efficient biomarkers in clinical application of detection. We use The Cancer Genome Atlas data as a training set and a multi-stage screening strategy to identify prognostic characteristics of melanoma based on DNA methylation. Three DNA methylation CpG sites were identified to be related to the overall survival in the skin cutaneous melanoma cohort. This signature was validated in two independent datasets from Gene Expression Omnibus. The stratified analysis by clinical stage, age, gender, and grade retained the statistical significance. The methylation signature was significantly correlated with immune cells and anti-tumor immune response. Moreover, gene expression corresponding to the candidate CpG locus was also significantly correlated with the survival rate of the patient. About 49% of the prognostic effects of methylation are mediated by affecting the expression of the corresponding genes. The prognostic characteristics of DNA methylation combined with clinical information provide a better prediction value tool for melanoma patients than the clinical information alone. However, more experiments are required to validate these findings. Overall, this signature presents a prospect of novel and wide-ranging applications for appropriate clinical adjuvant trails.

## Introduction

Cutaneous melanoma is one of the deadliest neoplasms with high aggressive and metastatic characteristics^1^. The favorable prognosis of melanoma is dependent of its timely detection in early stage^2^. Once the melanoma is established and developed, it becomes a threat to the patients’ life^3^. Therefore, the identification of novel effective prognostic indexes is needed to accelerate the detection of melanoma.

Aberrant DNA methylation is an epigenetic hallmark of cancer, which plays a significant role in cancer management^4^. It is increasingly recognized that epigenetic aberrations could induce cancer development and progression by inactivation of tumor suppressor genes (TSGs) at the promoter region^4^. Regarding melanoma, aberrant DNA methylation is the most widely studied dysregulated epigenetic mechanism^5^. Until now, over 100 genes contributing to melanoma pathogenesis have been identified to be aberrantly hypermethylated^5^. These genes include SOX9, MITF, TIM-3 and LGALS9^6–8^, which play critical roles in tumor cell biology after hypermethylation. The methylation patterns might be recognized as a biomarker for predicting the prognosis of patients. Furthermore, it is also reported that alterations of DNA methylation status can be involved in the tumor microenvironmental immune evasion and are able to modulate the response of immunotherapy^9^. Genomic methylation alterations counteract the contribution of high mutation burden and increase immunotherapeutic resistance^10^. Moreover, several epigenetic therapies have been developed inhibiting enzymes controlling epigenetic modifications^4^. For example, 5-azacytidine and 5-aza-2-deoxycytidine were discovered as promising inhibitors of DNA methylation for treating several malignancies^11^. Meanwhile, the use of inhibitors in combination with other key enzymes will greatly improve therapy and patient recovery^11^.

In the present study, we aim to construct a novel methylation biomarker signature for melanoma prognosis and further immunotherapy application. To do so, we have investigated the prevalence of CpG island methylation in TSGs silenced in cancer to design methylation profiles associated with main clinicopathological features. Moreover, we have explored the prognostic significance of these TSGs promoter methylations in melanoma outcomes. Comprehensive bioinformatics analyses were conducted to explore potential mechanism of biomarkers. This study presents an accurate predictive pattern for melanoma patients based on methylation and provides prospect of subsequent personalized medical care.

## Methods and material

### Data acquisition and processing

DNA methylation data (Illumina Infinium HumanMethylation450 array) from patients with skin cutaneous melanoma in the “cohort: GDC The Cancer Genome Atlas (TCGA) skin cutaneous melanoma (SKCM)” as training set were acquired from the Cancer Genomics Browser of The University of California Santa Cruz (UCSC) (https://xenabrowser.net/datapages/?cohort=GDC%20TCGA%20Melanoma%20(SKCM)&removeHub=https%3A%2F%2Fxena.treehouse.gi.ucsc.edu%3A443). RNA-sequencing data, protein expression data, and clinical information were also retrieved. The clinical data were preprocessed by exclusion of patients with missing follow-up information. Additional methylation data and corresponding clinical information for independent validation set 1 and set 2 were obtained from Gene Expression Omnibus (GEO) (196 patients, GEO accession number GSE144487^12^; 47 patients, GEO accession number: GSE51547^8^). All the data of methylation beta matrix was filtered and normalized with ChAMP R package^13^.

### Survival model construction process

**Figure 1:**
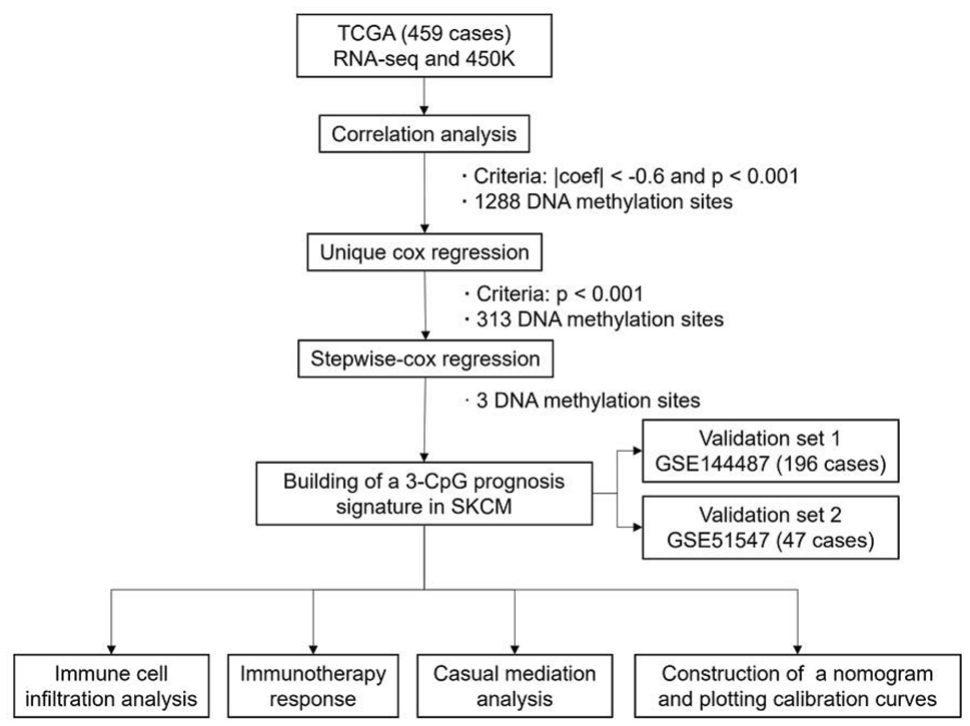
Flowchart showing steps involved in identification of the prognostic DNA methylation signature in melanoma.

At first, 459 tumor samples were included in our study by removing the samples with unavailable DNA methylation data, gene expression or survival data. The list of common CpG probes shared between 450K and EPIC array was selected to investigate the association between gene expression and DNA methylation across tumor samples. 342,127 DNA methylation sites were remains to follow-up filtration. Then, Pearson coefficient between gene expression and corresponding methylation β level in the promoter region (“1stExon”, “5'UTR”, “TSS1500”, “TSS200”) were calculated. A high negative Pearson coefficient <  − 0.6 with *P* < 0.05 was set as criteria for identification of expression related methylation probes. Subsequently, 1288 probes were identified to associated to expression and subjected to the unique Cox regression analysis. 313 probes were retained based on the criteria of *P* < 0.001. At last, the stepwise-cox regression analysis is used to construct a best fitting prognostic model. A risk score formula was established by considering the expression of optimized 3 probes measured by their estimated regression coefficients. Patients were classified into high or low risk groups with the median risk score as cutoff value. The study flowchart describing the process is shown in Fig. 1.

### Determination of tumor-infiltrating immune cells and immune responses

Cell-type identification by estimating relative subsets of known RNA transcripts (CIBERSORT), an analytical algorithm, is developed to offer an estimation of the abundances of 22 immune cell population in a mixed neoplasm tissue^14^. ESTIMATE predicts tumor purity, and the presence of infiltrating stromal/immune cells in tumor tissues^15^. The cytolytic activity (CYT) score was obtained by calculating the geometric mean of granzymes A (GZMA) and perforin (PRF1) mRNA expression levels in tissue^16^. RNAseq data was analyzed by edgeR algorithm and Gene Ontology (GO), KEGG pathway enrichment and Gene Set Enrichment Assay (GSEA) that were carried by clusterProfiler package.

### Tumor immune dysfunction and exclusion (TIDE) analysis

TIDE is a computational algorithm constructed by Jiang et al.^17^ to predict the immunotherapy response of immune checkpoint blockades. TIDE quantified the levels of two conventional mechanisms in the process of tumor immune evasion: T cell dysfunction and T cell exclusion. The TIDE score in patients with cutaneous melanoma from the TCGA cohort, including T cell dysfunction score and T cell exclusion score, were performed in the TIDE web (http://tide.dfci.harvard.edu) after uploading the transcriptome profiles. Then, the correlation among diverse immune parameters and signatures were evaluated.

### Mediation analysis

VanderWeele’s mediation analysis is widely applied to test a hypothetical causal chain^18^. In this case, the corresponding mRNA expression was regarded as mediators, and its indirect effect (HR_indirect_) was determined, standing for the regulation via mRNA expression. Meanwhile, the effect of methylation status on clinical prognosis is described as direct effect (HR_direct_). Finally, the whole effect of methylation on survival is called HR_total_, composed of the two variables, HR_indirect_ and HR_direct_.

### Nomogram construction

Nomogram was developed to predict the personalized estimation of the probability of prognosis, recurrence, or drug response^19^. In nomogram, each contributing parameter including age, gender, Tumor Node Metastasis stage (TNM stage), and risk score, was quantified by points and added up to generate a total point by individual. In the present study, we integrated the clinical characteristics and risk score in the nomogram for prediction of prognosis for each melanoma patients. The nomogram was processed via the rms package for R software. A calibration curve was applied to visualize the deviation between predicted probabilities and the actual situation, with 1000 replicates set up in the bootstrap method. The predictive accuracy of the nomogram was measured by the concordance index (C-index).

### Statistical analysis

Kaplan–Meier survival curves were drawn and compared among subgroups using log-rank tests to assess survival differences. Multivariate Cox regression and subgroups stratification analysis were performed to explore whether the methylation-based risk score was independent of patients’ clinical features. To evaluate the efficiency of the survival prediction among the risk score and the TNM stage, receiver operating characteristic (ROC) analysis was performed. Hazard ratios (HR) and 95% C-index were also calculated. *P* values were two-sided, and *P* < 0.05 was considered statistically significant. Statistical tests above were performed using R version 4.0.0 (The R Foundation).

### Ethics approval and consent to participate

Consent for participation for all patients was obtained through The Cancer Genome Atlas Project.

## Result

### Derivation of prognostic DNA methylation sites and construction of risk formula

**Figure 2:**
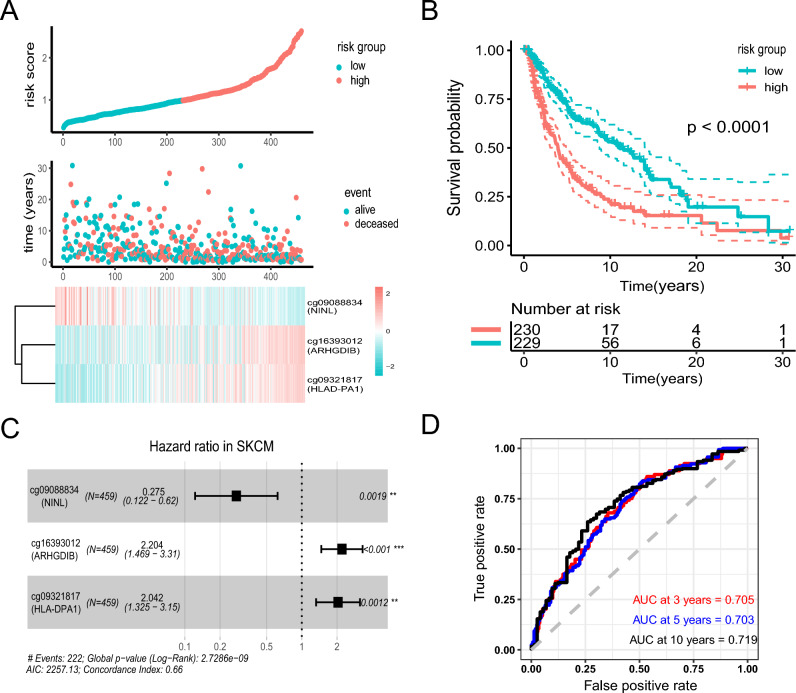
Construction of the 3-methylation signature in TCGA training set for determining survival outcomes. (**A**) The distribution of risk score, patients’ survival status and methylation expression panel. (**B**) Kaplan–Meier curves in all patients based on risk score. (**C**) 3 methylation probes were significantly correlated with overall survival derived from the univariable cox regression analysis in melanoma patients. (**D**) ROC analysis of 3 methylation for predicting of survival at 3, 5 and 10 years in TCGA cohorts.

The study was conducted on 459 patients in TCGA cohort who are clinically and pathologically diagnosed with cutaneous melanoma. Firstly, univariate cox proportional hazard regression analysis was carried to filter the candidate mRNA-related-DNA methylation level data in the training cohort. With the strict criteria (*P* < 0.001), a total of 313 DNA methylation sites were identified as candidate markers that significantly correlated with the overall survival (OS) of patients. Subsequently, these candidate sites were used to perform multivariate cox regression analysis with both-side stepwise. Finally, 3 methylation sites were selected to contribute one hazard ratio model as the optimum prognostic model for predicting OS. The liner risk score formula was created as follows: Risk score = (− 1.29) * β_cg09088834(NINL)_ + (0.7904946) * β_cg16393012(ARHGDIB)_ + (0.7137228) * β_cg09321817(HLA-DPA1)_. The detailed information of the three methylation probes is shown in Supplementary Table 1. The risk score for each patient was calculated using this formula. Then, patients were assigned into high-risk and low risk group based on the optimized risk value within the TCGA cohort (Fig. 2A). The survival of high-risk patients was significantly shorter than of low-risk patients in a Kaplan–Meier curve (Fig. 2B, *P* < 0.0001). The univariates Cox regression analysis was operated to evaluate the prognosis value of these sites respectively (Fig. 2C). Then, we calculated the time-dependent area under the ROC curves (AUC) to evaluate predictive performance. AUC values at 3-, 5- and 10-years indexes are 0.705, 0.703, and 0.719 respectively, representing a prognostic prediction (Fig. 2D).

### The signature is independent from clinical and pathological characteristics

**Figure 3:**
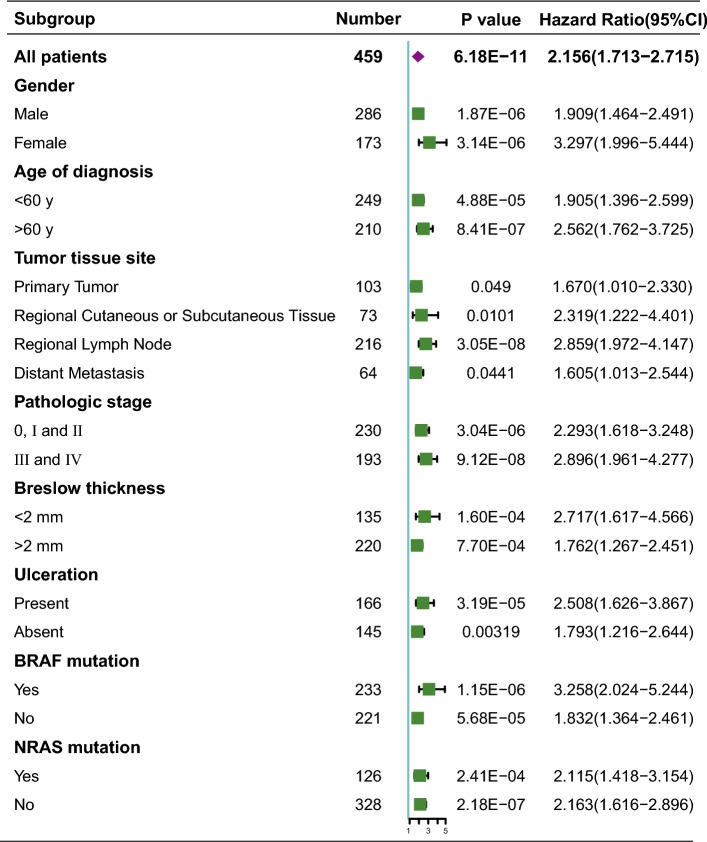
The prognostic value in subgroups based on clinicopathologic characteristics in TCGA cohort. The SKCM cohort was divided into subgroups based on clinicopathologic characteristics, such as gender, age of diagnosis, tumor tissue site, pathologic stage, Breslow thickness, ulceration, BRAF mutation and NRAS mutation.

There are varieties of clinical and pathological features that are considered as predictions for patients with melanoma, such as patients’ age, sex, AJCC stage, tumor thickness, the site where the sample was obtained, ulceration status, as well as mutation status^20–24^. Therefore, patients were regrouped according to different clinicopathological characteristics to assess the independence and applicability of this three-DNA methylation signature. Age, gender, tumor tissue site, pathologic stage, Breslow thickness, ulceration, BRAF and NRAS mutation status were taken into consideration. Consequently, the three-DNA methylation signature remains a good reference for different regrouped cohorts in the forest plot (Fig. 3). Meanwhile, patients with BRAF mutation shows a high-risk score comparing with counterparts without BRAF mutation. However, there is no difference about NRAS status (Supplementary Fig. 1). These results suggest that the methylation signature has a high effectiveness for risk stratification. The signature could therefore be used as an applicable independent prognostic predictor and guide clinicals to choose proper targeted drugs.

### Evaluation of the methylation signature for survival prediction in other independent cohorts

**Figure 4:**
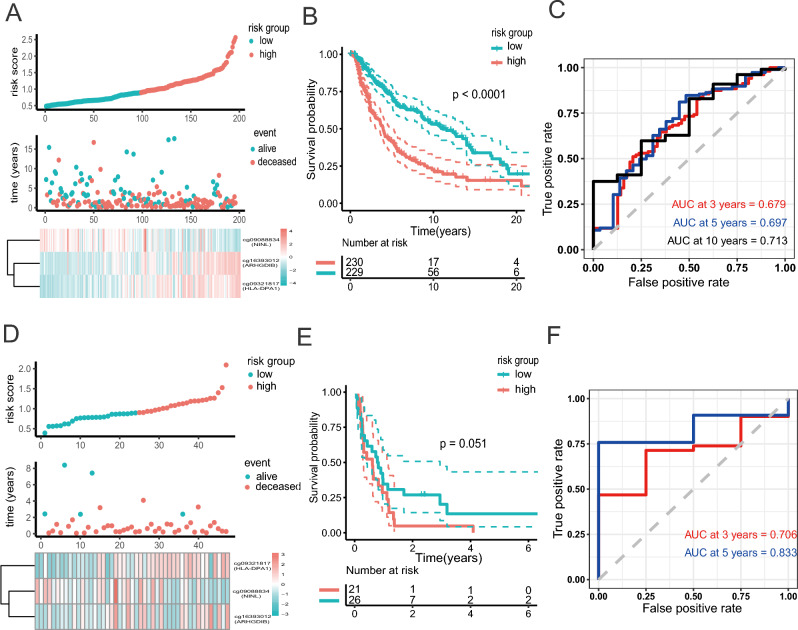
Validation of 3-methymation-signature by revealing the prognostic significance in the validation sets. (**A**–**C**). The distribution of risk score, patients’ survival status and methylation expression panel; Kaplan–Meier curves and ROC analysis for survival prediction in GSE144487. (**D**–**F**). The distribution of risk score, patients’ survival status and methylation expression panel; Kaplan–Meier curves and ROC analysis for survival prediction in GSE51547.

To further examine the prognostic values of the three-DNA methylation signature in another independent cohorts, Kaplan–Meier and ROC analyses were carried out in two other independent cohorts (GSE144487 N = 198; GSE51547, N = 47). Similarly, patients with high or low risk were grouped based on the median risk score of the training cohort (Fig. 4A, D). Patients in the low-risk group had a significantly longer overall survival in GSE144487, suggesting the three-DNA methylation performed well in prediction (*P* < 0.0001, Fig. 4B). 3-, 5-, and 10-year AUC were 0.678, 0.697 and 0.713, respectively (Fig. 4C). However, due to the limited sample size in the GSE51547 cohort, the log-Rank test was not significant but the AUC value in 3- and 5-year were 0.705 and 0.833 (Fig. 4E, F), confirming that the three-DNA methylation signature can also predict the survival of melanoma patients in other independent cohorts.

### Association between three-DNA methylation signature with immune cells and immune response

**Figure 5:**
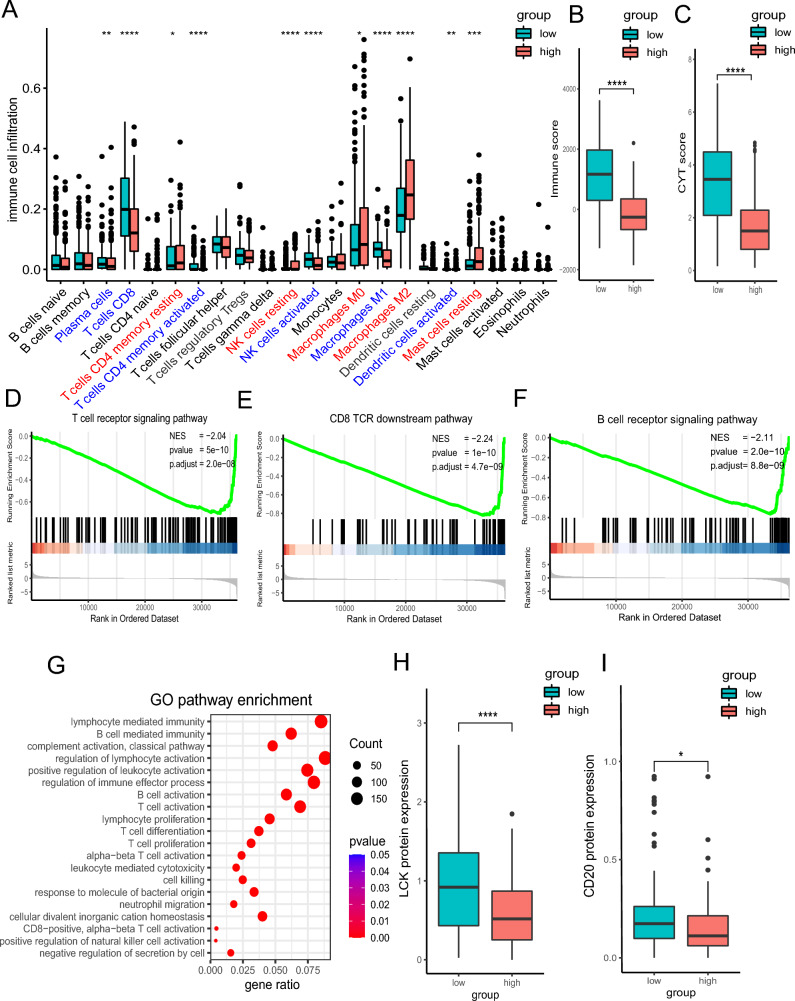
Analysis of composition of tumor infiltering leukocytes and immune response. (**A**) The comparison of 22 immune cell subsets between high score and low score groups based on CIBERSORT. (**B**) Estimated immune score comparison based on ESTIMATE. (**C**) CYT score comparison. (**D**–**F**). GSEA analysis of T cell receptor signaling pathway, CD8 TCR downstream pathway, and T cell receptor signaling pathway. (**G**) GO enrichment analysis. (**H**, **I**) Protein levels of LCK and CD20.

Immune cells calculated by CIBERSORT algorithm were compared in the two groups in the SKCM cohort. Diverse anti-tumor immune cells, including plasma B cells, CD8^+^ T cells, activated memory CD4^+^ T cells, activated natural killer (NK) cells, M1-like macrophages and activated dendritic cells (DCs) were found highly enriched in the low-risk population. Meanwhile, resting memory CD4^+^ T cells, resting NK cells, M0 macrophages, M2-like macrophages and resting mast cells were elevated in the high-risk population (Fig. 5A). Considering the role of TILs as an important marker, we analyze the prognostic value with this parameter. This signature may serve as a reliable independent prognostic predictor even when considering immune cells as covariates (Supplementary Fig. 2). Immune score calculated by ESTIMATE algorithm was higher in the risk-low group (Fig. 5B). The cytolytic activity (CYT) score is a new index of cancer immunity calculated from the mRNA expression levels of GZMA and PRF1, representing immune cytotoxicity^16^. CYT score was higher in risk-low group (Fig. 5C). Moreover, GSEA analysis GSEA analysis was performed by comparing the risk-high and risk-low group, uncovering that T cell receptor (TCR), CD8^+^ TCR downstream and B cell receptor pathways were significantly downregulated in the high-risk group (NES = − 0.24, *p* = 2.0e−6; NES = − 0.24, *p* = 2.0e−6; NES = − 0.24, p = 2.0e−6; Fig. 5D–F). Gene Ontology (GO) enrichment analysis showed that immune response related pathways, including T cell receptor signaling pathway, CD8 TCR downstream pathway, and T cell receptor signaling pathway, were also highly enriched in the low-risk group (Fig. 5G). Finally, in Reverse Phase Protein Array (RPPA) of SKCM cohorts, the protein levels of LCK and CD20 were higher in low-risk group, suggesting a boost of the anti-tumor immune response (Fig. 5H, I).

### Relationship between the methylation-based signature and immunotherapy response

**Figure 6:**
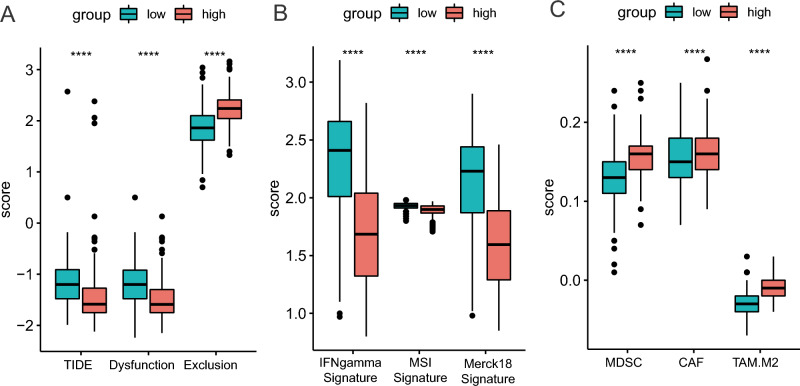
Distribution of immunotherapy response markers in high- and low-risk groups. (**A**) The distribution of TIDE score, T cell dysfunction score, and T cell exclusion score in high- and low-risk groups. (**B**) The distribution of MSI signature, Merk18 signature, and IFN signature in high- and low-risk groups. (**C**) The distribution of MDSC, CAF and TAM in high- and low-risk groups. *, **, *** and **** represent *P* < 0.05, *P* < 0.01, *P* < 0.001 and *P* < 0.0001, respectively.

Next, in order to predict the response of immunotherapy, we introduced the TIDE analysis into our study^17^. As expected, patients in the high-risk group displayed a significantly lower TIDE score, as well as T cell dysfunction (Fig. 6A). However, the high-risk population is characterized by higher exclusion scores (Fig. 6A). It is also widely accepted that microsatellite instability could be a predictor for immunotherapy efficiency^25^. MSI signature, a prediction model based on ridge regression, was higher in the low-risk group as compared to the high-risk group (Fig. 6B). Merk18 expression is considered as a T-cell inflamed signature for pan-cancer predictors of clinical benefit from anti-programmed cell death Protein 1 (PD-1) treatment^26^. Whereas IFN-γ-related mRNA profile predicts clinical response to PD-1 blockade^27^. Both Merk18 and IFN-γ-signature were elevated in the low-risk group (Fig. 6B). Finally, we investigated myeloid-derived suppressor cells (MDSC), cancer associated fibroblasts (CAF) and tumor-associated macrophages (TAM) in the tumor suppressive immune microenvironment. All of these are well-known biomarkers to predict the response of immunotherapy. We found higher MDSC, CAF and TAM levels in high-risk patients (Fig. 6C). These results suggest that the CpG-based signature could highlight a population with low-risk patients who may be more responsive to immunotherapy, especially immune checkpoint blockage (ICB).

### The underlying mediation of mRNA expression in the effect of methylation on overall survival

**Figure 7:**
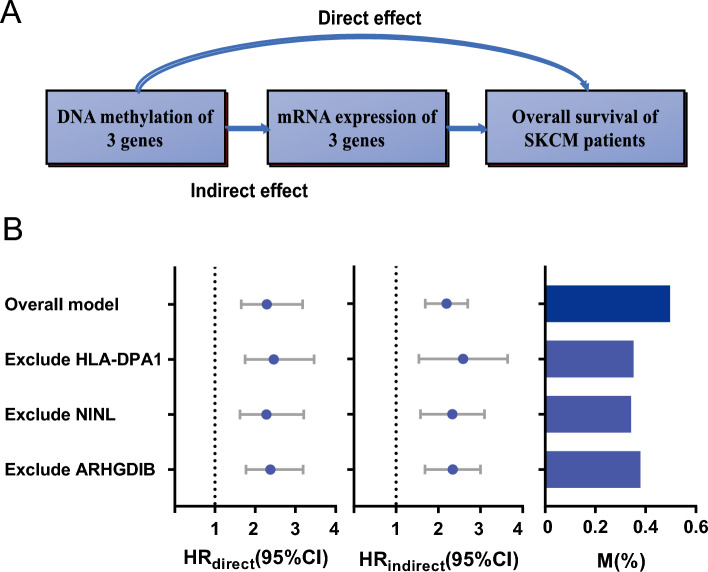
The mediation analysis for methylation prognostic signature through mRNA expression. (**A**) Diagram of mediation model. (**B**) The 3 CpG sites were treated as “exposure”; mediator was the linear combination of the corresponding 3 mRNA expression level (score_exp_) in overall model. Prognostic effect in hazard ratio (HR) were described as direct effect (HR_direct_) and indirect effect (HR_indirect_), corresponding 95% confidence interval (95% CI), and the proportion of effect mediated (M%). Further, sensitivity analyses were performed by excluding each gene from score_exp_, respectively.

To illustrate the underlying mediation pathway between methylation, and overall survival, the VanderWeele’s mediation analysis was performed. In this overall mediation model, the mediator was risk score calculated by linear combination including three genes’ mRNA expression (Fig. 7A). There is a clear relation between the methylation status and the corresponding mRNA expression level for the three CpG sites (Supplementary Fig. 3). Furthermore, these 3 genes were also associated with patients’ outcomes (Supplementary Fig. 4). Subsequently, prognostic equation based on the mRNA expression was also performed as described above: score_expression_ = (0.10) * NINL + (− 0.05) * ARHGDIB + (− 0.14) * HLADPA1. Interestingly, this score was significantly associated with the prognosis. In the well-constructed mediation model, HR_indirect_ is 2.15 (95% CI: 1.71–2.72; *P* < 0.0001). The methylation signature was highly mediated via affecting the respective mRNA expression to predict the survival outcomes of patients (proportion mediated, 49%; Fig. 7B). To avoid bias in the findings, sensitivity analysis was performed by excluding each gene expression from score_expression_. We were able to find that the consequence of the mediation role of mRNA expression remained statistically significant (Fig. 7B).

### Construction of a predictive nomogram

**Figure 8:**
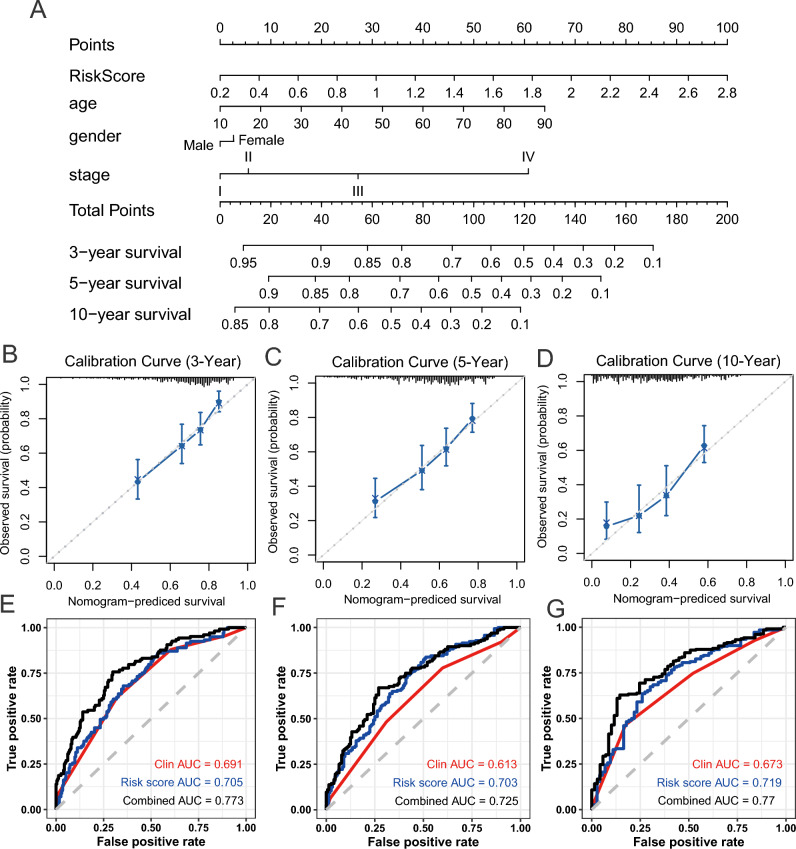
Nomogram and Calibration plot. (**A**) Nomogram to predict the 3-, 5- and 10-year OS. (**B**–**D**) Calibration curve for OS nomogram model in 3-, 5- and 10-year. The dot line represents the ideal nomogram, and the blue line represents the observed nomogram. (**E**–**G**) ROC analysis of the sensitivity and specificity of OS prediction by the DNA methylation signature risk score, clinical information, and combination of the two factors.

To develop a clinically applicable method predicting an individual’s outcome probability, we used a nomogram to build a predictive model, taking into consideration clinicopathologic covariates. Considering the basis of the multivariate analysis of overall survival, we generated a nomogram to predict the 3-, 5-, and 10-year OS in the training cohort (Fig. 8A). The predictors included age, gender and TNM stage. The calibration plots for the 3-, 5-, and 10-year OS rate were successfully predicted (Fig. 8B–D). Combination of clinical information, methylation data (AUC = 0.773, 0.725, and 0.77 for 3-, 5- and 10-year) showed a superior prediction ability in comparison to the model using clinical data only (AUC = 0.691, 0.613, and 0.673 for 3-, 5- and 10-year; Fig. 8E–G).

## Discussion

In recent years, the importance of DNA methylation in the biology of cutaneous melanoma has been increasingly appreciated^5^. For instance, it is well described that tumor suppressor gene promoters display the focal DNA hypermethylation in many cancers, including melanoma^5^. Furthermore, methylation features could be used to classify distinct subgroups with differing survival outcomes and biologic behavior within melanoma patient cohorts^28^. Previous studies discovered that DNA methylation regulates the expression of key genes such as tyrosinase (TYR), tyrosinase-related protein 1 (TYRP1), dopachrome tautomerase (DCT) and microphthalmia-associated transcription factor (MITF), as well as paracrine factors such as stem cell factor (SCF) and endothelin-1 (ET-1) in melanogenesis, a very important process in both cutaneous and ocular melanoma biology^29–32^. Previous study have revealed that a "Yin and Yang" role for melanin and active melanogenesis in melanoma development, progression, and therapy^33^. However, these studies usually concentrated on single gene methylation, which were unable to achieve a good performance of prediction. Moreover, their application was limited to specific clinical characteristics due to small patient numbers. Consequently, compared with individual DNA methylation, a combination of DNA methylation as biomarkers could achieve a better sensitivity and specificity as predictive pattern^34^.

This signature is confirmed by its high reproducibility and utility in various clinical groups with potential clinical applicability. Cutaneous melanoma has a high heterogeneity in terms of genome, clinical features, and histopathological characteristics^35^. Several parameters, including age, gender, stage of disease, Breslow thickness, and ulceration status, have significant influence on melanoma patient prognosis, relapse, and therapy responses^23^. For instance, the methylation signature could present reliable independence of the clinical factors mentioned above in regard to utilization. BRAF and NRAS are mutant genes in skin cutaneous melanoma associated with different tumor immune microenvironments^24^. Thus, we analyzed the performance of the signature among patients with different BRAF and NRAS mutation status. We could demonstrate that our signature is independent of these mutations and could be applicable as a prognostic biomarker. Meanwhile, considering that an ideal prognostic marker could efficiently stratify in other independent cohorts, we employed two other GEO datasets (GSE14487 and GSE51547) to validate the practicality of our three-DNA methylation signature. The 3-DNA methylation signature performed well in distinguishing low-risk and high-risk groups in these two independent cohorts as well, suggesting that this signature may be of high clinical value with wide-ranging applications.

In the process of anti-tumor response, immune cells infiltrate into tumor tissue. The tumor cells work as antigens and activate the host immunity system^36^. The grade of tumor-infiltrating lymphocytes is an independent protective factor, which displays an excellent prognostic marker in melanoma^37^. Meanwhile, immune checkpoints targeted therapy has facilitated great progress in treating multiple cancers. It is well known that immune inhibitors including PD-1, programmed death-ligand 1 (PD-L1), and cytotoxic T-lymphocyte-associated Protein 4 (CTLA-4) enable patients to produce an effective antitumor response, especially in melanoma^38^. However, there is a limitation that only one-third of patients show clinical benefit from immunotherapy^39^. Given the present situation, methylation status in DNA promoters play a critical role in cell lineage specification. Therefore, we hypothesize that DNA methylation signature may serve as a specific molecular predictor for the evaluation of immune activity^40^. To preliminarily assess the predictive ability of the methylation-based signature, six different well-validated immunotherapy biomarkers were used. The TIDE score was created to serve as an accurate biomarker for the immune checkpoint blockade response^17^. Microsatellite instability has been reported as a predictive factor for immunotherapy in malignant melanoma^25^. Merk18 expression represents a T cell inflamed signature that predicts therapeutic efficacy in patients treated with pembrolizumab across 20 cancers^26^. IFN-γ signature predicts clinical response to PD-1 blockade^27^. MDSCs, CAFs and TAMs are well known classic biomarkers of the response to anti-PD-1/PD-L1 therapies^41–43^. We found that high-risk patients had significantly lower levels of TIDE score, MSI, Merk18, IFNγ-signature, MDSCs, CAFs and TAMs. In the current study, these novel findings represent that our methylation-based signature, although developed for accurate prognosis, also significantly correlates with immune cell infiltration and anti-tumor immune response. However, further studies are needed to verify the ability of this signature to predict immunotherapy response.

In addition to DNA methylation, mRNA expression levels of three genes also affected prognosis significantly. NINL is identified as an oncogenic protein which causes spontaneous tumorigenesis in transgenic mice^44^. ARHGDIB is a metastasis suppressor gene affecting the migration of T cells in varieties of cancers^45,46^. HLA-DPA1 is mostly expressed in antigen presenting cells and plays a central role in the immune system by presenting peptides derived from extracellular proteins^47^. Mediation analysis showed that around 49% of the methylation prognostic effect is mediated through affecting corresponding gene expression. Meanwhile, the mediational role of transcriptional regulation is confirmed by sensitivity analysis.

At last, we built a nomogram including methylation score and clinical pathology to predict individual prognosis. Prognostic signature integrating DNA methylation and clinical information provides a better prognostic prediction value for melanoma patients than clinical information only. Our nomogram provides a simple and accurate prognostic tool for OS indication for patients with melanoma.

The study’s limitations should be noted. Firstly, the biologic mechanisms of the candidate markers NINL, ARHGDIB, and HLADPA1 are still unknown. Secondly, although this study was validated using two independent cohorts, clinical investigations in different populations are needed to validate the prognostic value of this signature. Last but not least, wet-lab experiments are required to further prove the correlation with the immune cells and the anti-tumor immune response.

## Conclusion

In this study, we constructed a powerful DNA methylation signature with a high predictive efficiency for prognosis in melanoma. The identified signature is independent from clinical and pathological characteristics and had a good performance in multiple cohorts. In the future, we hope that this signature could be used to refine the current prognostic model and to optimize the treatment strategy for patients with melanoma.

### Supplementary Information

Supplementary Information.

## Data Availability

Clinical information, high-throughput sequencing-counts, and DNA methylation data were retrieved from the UCSC xena (https://xenabrowser.net/datapages/?cohort=GDC%20TCGA%20Melanoma%20(SKCM)&removeHub=https%3A%2F%2Fxena.treehouse.gi.ucsc.edu%3A443), which is a publicly available database.

## Abbreviations

TCGA: The Cancer Genome Atlas
GEO: Gene Expression Omnibus
SKCM: Skin cutaneous melanoma
TSG: Tumor suppressor genes
UCSC: University of California Santa Cruz
CIBERSORT: Cell-type identification by estimating relative subsets of known RNA transcripts
GZMA: Granzymes A
PRF1: Perforin
GO: Gene ontology
GSEA: Gene Set Enrichment Assay
TIDE: Tumor Immune Dysfunction and Exclusion
SOX9: SRY-Box Transcription Factor 9
MITF: Melanocyte Inducing Transcription Factor
TIM-3: T-Cell Immunoglobulin Mucin Receptor 3
LGALS9: Galectin 9
MDSC: Myeloid derived suppressor cell
CAF: Cancer associated fibroblast
TAM: Tumor-associated macrophage
