# Supplementary material for: Identification of a CpG-based signature coupled with gene expression as prognostic indicators for melanoma: a preliminary study
Source: Sci Rep. 2024 Mar 4;14:5302. doi: 10.1038/s41598-023-50614-2 (PMC10912562; doi:10.1038/s41598-023-50614-2)
Supplement: Supplementary file 1 — Supplementary Information. [file 41598_2023_50614_MOESM1_ESM.docx]

Supplementary Data:


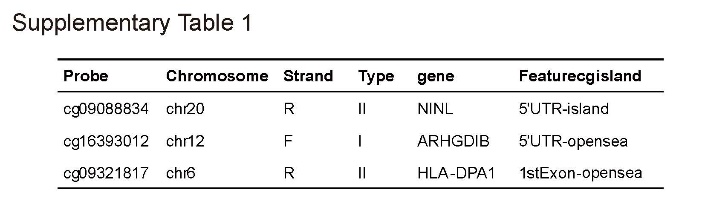


**Supplementary Table 1:** Three dividual methylation probes associated with overall survival of melanoma patients.


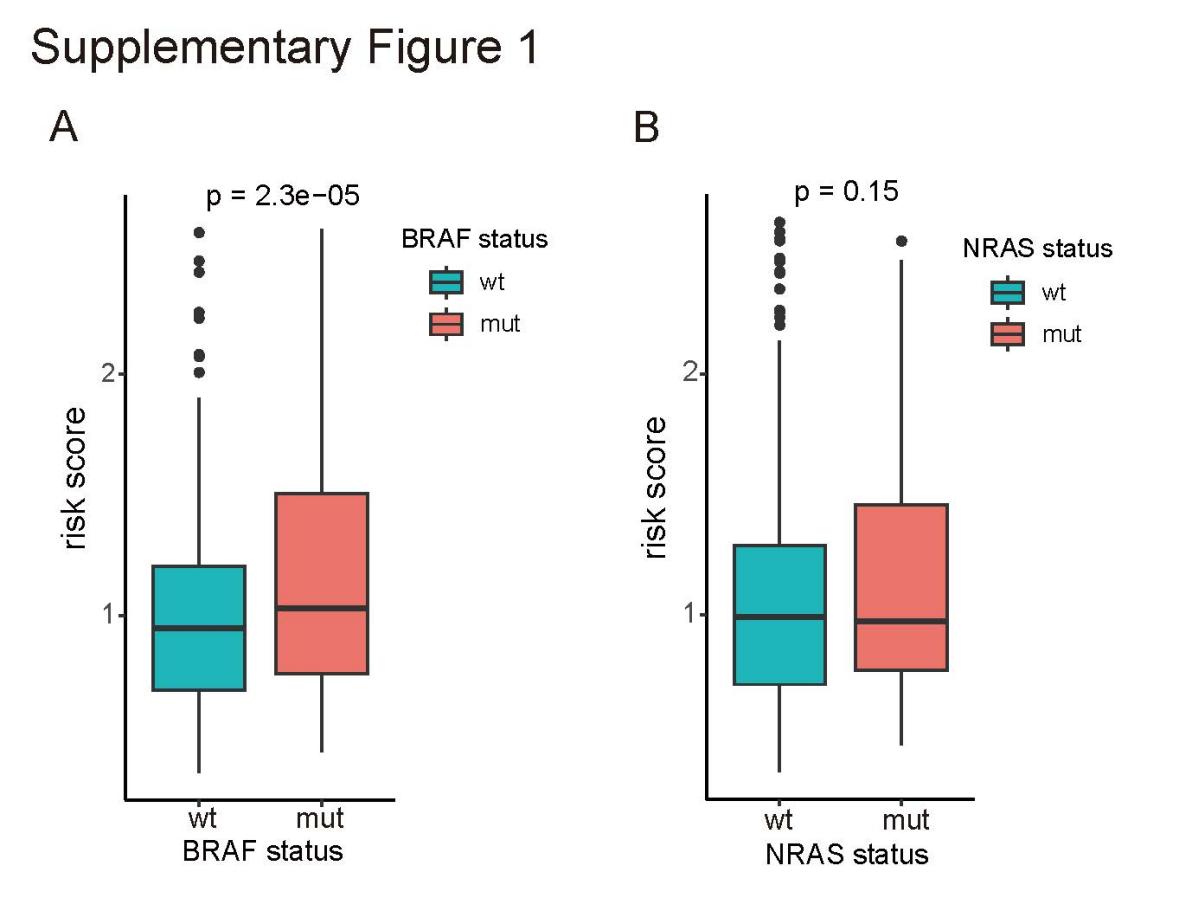


**Supplementary Figure 1:** The distribution of methylation-based signature in different BRAF status(A) and NRAS status(B).


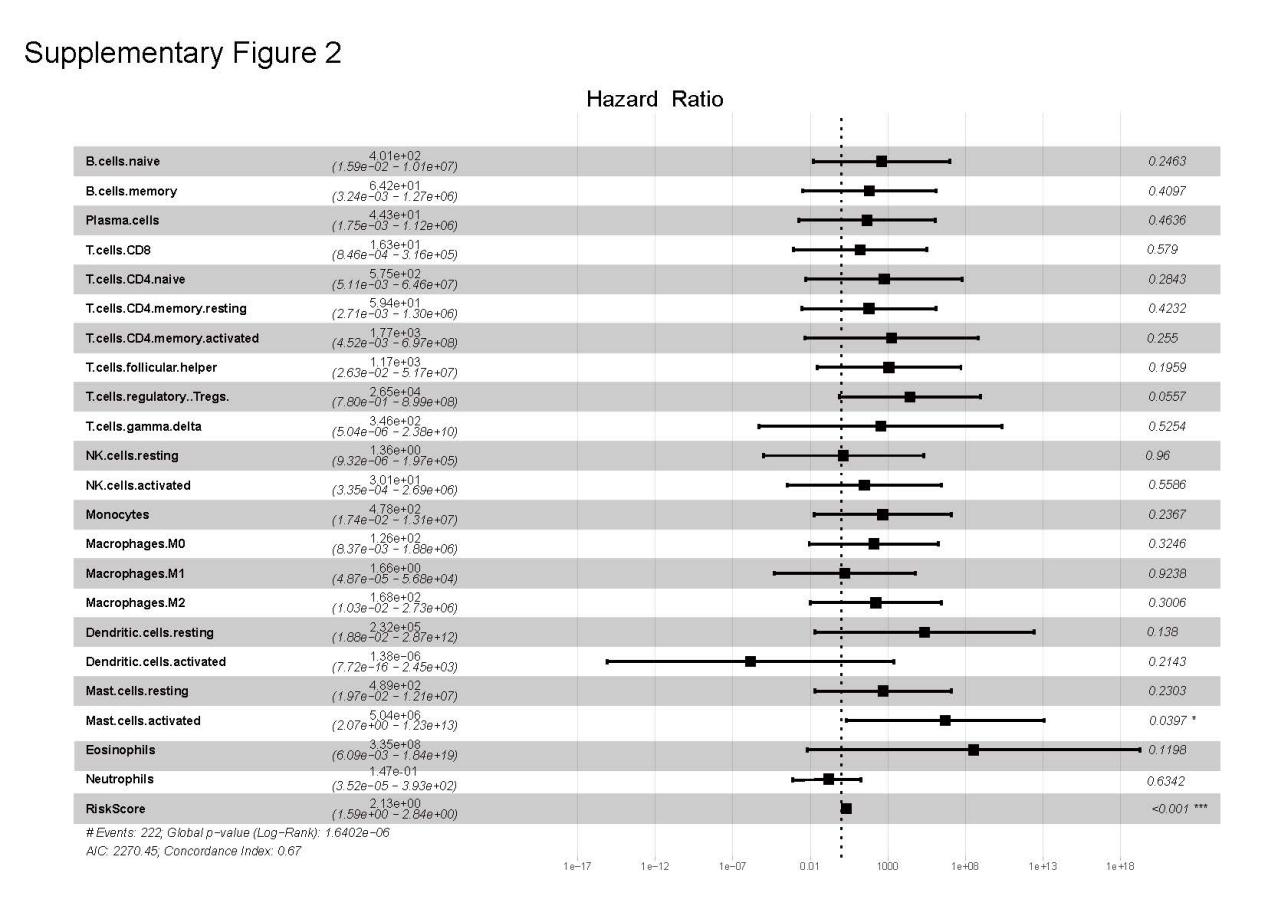


**Supplementary Figure 2:** The multi-cox regression analysis was performed considering 22 immune cells as covariates.


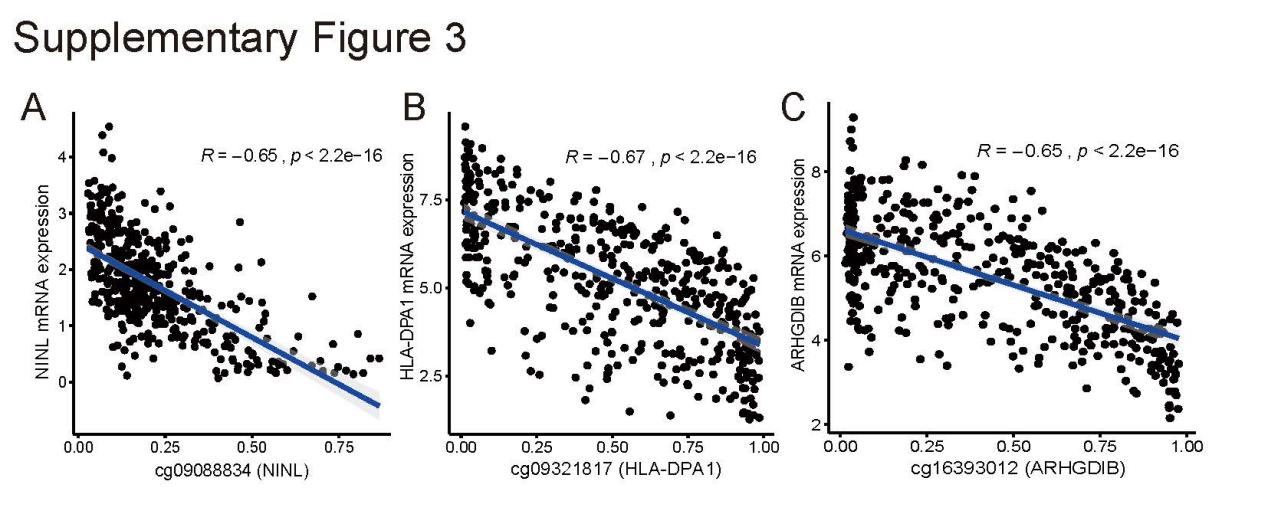


**Supplementary Figure 3:** Correlation between methylation and mRNA expression of cg09088834(NINL, A), cg09321817(HLADPA1, B) and cg16393012(ARHGDIB, C).


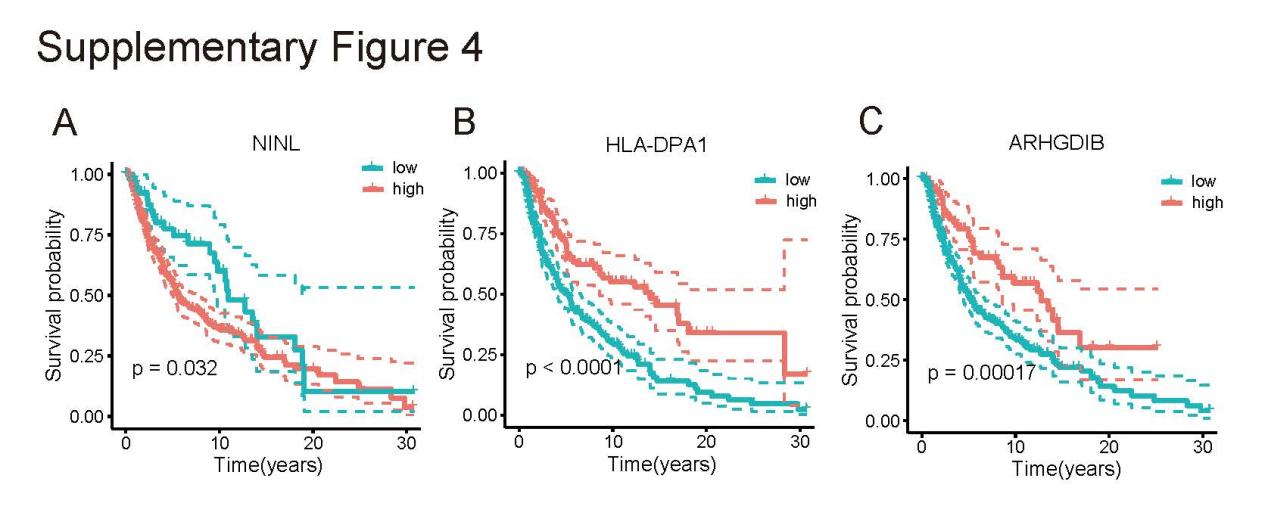


**Supplementary Figure 4:** Survival curve for 3 corresponding mRNA, NINL(A), HLA-DPA1(B), and ARHGDIB(C).
